# Teasing Out the Effects of Latitude and Birth Date on Allergy

**DOI:** 10.1371/journal.pmed.0020375

**Published:** 2005-10-04

The prevalence of asthma and allergy, defined as immunologically mediated hypersensitivity, is increasing. It is estimated that more than 20% of the world's population has IgE-mediated allergic diseases. The scale of the clinical problem is immense. The World Health Organization estimates that asthma affects nearly 150 million people worldwide, and more than 180,000 deaths each year are due to asthma. Approximately US$20 billion is spent globally each year on allergic rhinitis, including medications, time off work, and clinician consultations. The cost of allergy drugs alone is estimated to be US$8 billion per annum.

While allergy prevalence is increasing, the causal risk factors are still unknown. Matthias Wjst and colleagues investigated whether the spatial (latitude) and temporal (birth month) distribution of risk factors might offer insight into the mechanism of disease.

Previous studies have already shown that birth month is a risk factor associated with allergy; as the authors point out, birth month, used as a proxy for early allergen exposure, might be associated with upper respiratory infections during winter months. Studies have also associated geographical latitude with allergy. But some experts have noted that latitude, a proxy for ultraviolet solar exposure, might also reflect climatic differences, genetic influences, or even cultural differences in the raising of children.

In this study, Wjst and colleagues tried to further understand the effects of latitude and birth date on the prevalence of allergy defined by markers such as allergic rhinitis, sensitization to grass or dust, and total IgE levels. They distributed a questionnaire to 20– to 44–year-old individuals in 54 centers across Europe, North Africa, India, North America, Australia, and New Zealand. Altogether, data from 200,682 participants were analyzed. [Fig pmed-0020375-g001]

**Figure pmed-0020375-g001:**
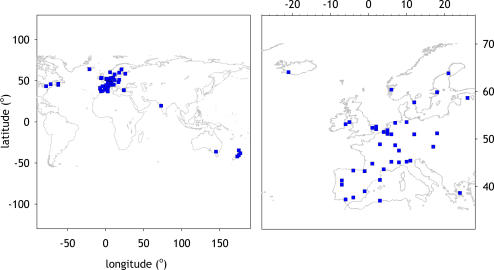
World and European distribution of study centres

The median prevalence of allergic rhinitis was 22%, but with a substantial variation across centers. They found allergic rhinitis decreased with geographical latitude, but there were many exceptions. There was no increase in prevalence during certain winters, and no altered risk by birth month, except borderline reduced risks in September or October. Altogether, the authors concluded that there was no major risk by being born in a particular month or during a particular season. There may be relevant birth month effects in single centers, but a global effect was questionable.

Previous research on the effect of birth month has also shown mixed results: differing studies have found associations that were positive, negative, or simply unclear.

But the authors noted that one difference of their study compared with others was the higher age of the subjects—interviewees were born between 1945 and 1973—and suggested that it might be possible that there are more marked symptoms in children that were being lost in adulthood.

Most previous studies have shown an association with allergic sensitization, indicating subclinical effects that gained importance only when occurring in combination with additional risk factors. One of the main advantages of this study—a standardized allergen test protocol—might, thus, be a disadvantage since the effects of local allergens might have been missed. Another methodological restriction might have been the use of self-reported “hay fever.” This term may be used in a different way across Europe, said the authors.

Data on the geographical distribution of allergic diseases are rare, and, hence, this study is valuable. However, a previous meta-analysis has shown negative association of latitude and symptoms of allergic rhinitis, with a −0.05% decrease per degree. In this study, symptoms of allergic rhinitis decreased with geographical latitude on a worldwide scale, but not when the analysis was restricted to Europe alone. One intriguing possibility, which needs further work, is that a risk factor within language borders might be more relevant than geographical latitude alone in determining the distribution of allergic diseases.

